# Investigating the Influence of Temperature on the Kaolinite-Base Synthesis of Zeolite and Urease Immobilization for the Potential Fabrication of Electrochemical Urea Biosensors

**DOI:** 10.3390/s17081831

**Published:** 2017-08-08

**Authors:** David Ebo Anderson, Srinivasan Balapangu, Heidimarie N. A. Fleischer, Ruth A. Viade, Francis D. Krampa, Prosper Kanyong, Gordon A. Awandare, Elvis K. Tiburu

**Affiliations:** 1 Department of Biomedical Engineering, University of Ghana, P. O. Box LG 25, Legon, Accra, Ghana; andersondavidebo@gmail.com (D.E.A.); srinivasan_bs85@yahoo.com (S.B.); 369hmnaf@gmail.com (H.N.A.F.); ruthviade@gmail.com (R.A.V.); 2 School of Engineering, Ulster University, Jordanstown BT37 0QB, UK; p.kanyong@waccbip.org; 3 Department of Biochemistry, Cell and Molecular Biology, University of Ghana, P. O. Box LG 25, Legon, Accra, Ghana; gawandare@hotmail.com; 4 West African Centre for Cell Biology of Infectious Pathogens, University of Ghana, P. O. Box LG 25, Legon, Accra, Ghana; fkrampa@gmail.com

**Keywords:** zeolites, electrochemical, urea, biosensor, nanoparticles

## Abstract

Temperature-dependent zeolite synthesis has revealed a unique surface morphology, surface area and pore size which influence the immobilization of urease on gold electrode supports for biosensor fabrication. XRD characterization has identified zeolite X (Na) at all crystallization temperatures tested. However, N_2_ adsorption and desorption results showed a pore size and pore volume of zeolite X (Na) 60 °C, zeolite X (Na) 70 °C and zeolite X (Na) 90 °C to range from 1.92 nm to 2.45 nm and 0.012 cm^3^/g to 0.061 cm^3^/g, respectively, with no significant differences. The specific surface area of zeolite X (Na) at 60, 70 and 90 °C was 64 m^2^/g, 67 m^2^/g and 113 m^2^/g, respectively. The pore size, specific surface area and pore volumes of zeolite X (Na) 80 °C and zeolite X (Na) 100 °C were dramatically increased to 4.21 nm, 295 m^2^/g, 0.762 cm^3^/g and 4.92 nm, 389 m^2^/g, 0.837 cm^3^/g, in that order. The analytical performance of adsorbed urease on zeolite X (Na) surface was also investigated using cyclic voltammetry measurements, and the results showed distinct cathodic and anodic peaks by zeolite X (Na) 80 °C and zeolite X (Na) 100 °C. These zeolites’ molar conductance was measured as a function of urea concentration and gave an average polynomial regression fit of 0.948. The findings in this study suggest that certain physicochemical properties, such as crystallization temperature and pH, are critical parameters for improving the morphological properties of zeolites synthesized from natural sources for various biomedical applications.

## 1. Introduction

A biosensor is an analytical device capable of producing a measurable signal as a result of the interactions between a biological agent, such as an enzyme, and its substrate; they include amperometric (current based), potentiometric (voltage based), and conductometric biosensors. The electrochemical-based sensors have been used for years, due to their lower dependence on solution turbidity and their relatively simple architecture [1]. The set-up usually consists of a bio-recognition element, which is attached to a working electrode or a transducer, which offers selectivity for its substrate. However, the biosensor layout of the early generation has several limitations, including the leaching out of the biomolecule, resulting in narrow linear range and low sensitivity. In enzyme-based biosensors, enzyme immobilization on a suitable support is a key step in achieving an improved sensitivity in the biosensors. Thus, methods to improve a higher surface area-to-volume ratio, which influences the size and shape of the pore of the material and its bearing on the electrochemical properties of the biosensor, is a fundamental requirement.

The sensitivity and overall performance of enzymatic biosensors has improved tremendously over the years as a result of the synthesis and incorporation of nanomaterials in their fabrication [2,3,4,5]. These materials have excited engineers due to their unique structural, electrical, chemical and mechanical properties [6,7,8,9]. In the field of the fabrication of medical devices, there are currently numerous studies describing the use of gold nanoparticles, graphene, zeolites, quantum dots, silver nanomaterials, and nanocomposites, as well as carbon nanotubes (CNTs) for enzyme immobilization, due to certain characteristics ranging from their biocompatibility, fast response times and the ease of modifying their features [1,2,3,4,5,6,7,8,9,10,11,12,13]. One of the oldest known materials, for example, are clay minerals which have been used for curative, industrial and medical applications which have predominated for several centuries [14,15]. They have been widely applied in such fields because of their high colloidal and swelling capacity, surface reactivity, high adsorption and cation exchange capacity [16,17,18]. Some of the most studied biosensors are those developed for urea detection, using the different sensing methods mentioned [19,20,21,22,23]. Briefly, urea is an organic molecule with the chemical formula CO(NH_2_)_2_ and is the end-product of protein metabolism in the liver. Urea undergoes filtration and a series of processing activities before it is finally excreted with urine; it is an important biomarker for detecting renal dysfunction using different testing methods, including biosensors. As a well-studied model system, urea biosensors function on the basis that urea is decomposed by urease to form electroactive species (ammonium ion) accruing to the reaction:

(NH_2_)_2_CO + 2H_2_O → 2NH_4_^+^ + HCO_3_^-^ (1)

The electrochemical monitoring of the enzymatically produced NH_4_^+^ at an appropriate transducer generates the analytical response.

There are currently concerted efforts by the scientific community tailored towards identifying materials that can enhance enzyme immobilization, because of the high demand in the design of biosensors for rapid testing at point-of-care.

Zeolites are aluminosilicates with interconnected microspores which contain countercations. Because of the pore network, zeolites have been used for immobilizing urease on working electrodes for urea and other bio-sensing applications; these includes zeolite BEA, Zeolite Na A [24,25,26]. The types of zeolites formed are a function of temperature, pressure, concentration of the reagent solutions, the pH, the process of activation and ageing period, and the SiO_2_ and Al_2_O_3_ contents of the raw materials. A contribution from the current study to the already existing zeolite-based biosensors is to synthesize different zeolites from kaolin and to test their usefulness for enzyme immobilization for various biological applications. Studies have also correlated the dependence of the responses of urea biosensors to ionic strength and have shown that decreasing sensitivity is achieved with increasing ionic strength. Thus, taking advantage of diverse elements such as sodium and potassium in natural zeolites, which often tend to neutralize the net charge on the zeolitic material, this work also seeks to compare the response of the zeolite modified electrode (ZME) with that of the bare electrode.

## 2. Material and Approach

### 2.1. Materials and Equipment

Kaolin deposits from Amanfrom in the Eastern part of Ghana were collected and purified (coordinates: 6.2374° N, 0.4502° W). Sodium Hydroxide (NaOH) pellets (99% pure) were obtained from (Sigma-Aldrich, St. Louis, MO, USA). Deionized Milli-Q distilled water was purchased from (Millipore, Bedford, MA, USA). The urease enzyme was obtained from (Urease active meal, from Jack beans). The urea stock was of domestic production, from the department of chemistry at the University of Ghana, Phosphate Buffered Saline, pH 7.4 (Sigma Aldrich, St. Louis, MO, USA). The gold electrode was from Griffin; the magnetic stirrer and hot plate from IKAMAG RCT (IKA, Germany); the furnace from Thermo-Scientific (Waltham, MA, USA) and Metrohm AutoLab PGSTAT 204 (Metrohm Autolab, AC Schiedam, Netherlands). SEM (JSM-7100F, Scanning Electron Microscopy, JOEL, Waltham, MA, USA), EDX (JEOL JSM-7100F equipped with Thermo Scientific UltraDry EDX, Waltham, MA, USA), and XRD (PANAnalytical diffractometer, Almelo, Netherlands).

### 2.2. Zeolite Synthesis

The zeolite synthesis was carried out using a combined approach involving alkaline fusion before hydrothermal reaction [27,28]. Briefly, a known amount of kaolin was ground into granules and sieved with 63 μm molecular cut-off sieve, and the particles were calcined at 650 °C in a furnace for 2 h to obtain a more reactive kaolin (Metakaolin) using a crucible. The metakaolin was allowed to cool and was mixed with NaOH powder in a mass ratio of 2:3 (% weight per weight, *w/w*) in a crucible. The ensuing mixture was fused at 600 °C for 2 h in a furnace, and the fused product was crushed into fine powder in a mortar to increase its reactivity with distilled water. The resulting mixture was added to distilled water in a ratio of 1:5 (*w/w*), stirred for about 1 h for gelation, after which it was left at room temperature for additional 24 h for aging inside a beaker. The beaker with the gel was kept in an oven for crystallization at various temperatures (60 °C, 70 °C, 80 °C, 90 °C, and 100 °C) for 24 h. After crystallization, the solid part was separated by low speed (1000 rpm) centrifugation before washing with distilled water to drop the pH from 13.6 to 7.5–8.0, and dried for 12 h in a hot air oven set at 60 °C to obtain fine powder. Note, this synthetic route is common to most zeolites but the final product depends on the base material.

### 2.3. Zeolite Characterization

XRD was performed with a PanAnalytical diffractometer using CuKα radiation at 2θ, scanning from 5 to 50 degrees in steps of 0.05 degrees, with a tube voltage of 45 kV and a current of 40 mA. X-ray powder diffraction (XRD) results confirmed zeolite X (Na) was obtained from the synthesis. The dehydration process did not affect the crystal nature of the zeolite X (Na) obtained. Zeolite X (Na) crystallised at different temperatures were labelled as follows; zeolite X (Na) 60 °C, zeolite X (Na) 70 °C, zeolite X (Na) 80 °C, zeolite X (Na) 90 °C, zeolite X (Na) 100 °C. EDX spectra and the elemental composition of each sample were obtained at an accelerating voltage of 10 kV using a JEOL JSM-7100F field emission scanning electron microscope equipped with a thermo scientific UltraDry EDX detector. Each sample was then removed from the scanning electron microscope (SEM) machine after the EDX analysis and sputter-coated with 3 nm of gold. SEM micrographs were obtained at an accelerating voltage of 10 kV using a JEOL JSM-7100F field emission scanning electron microscope. Approximately 50 μm of colloidal graphite (Agar Scientific Ltd., Stansted, UK) was uniformly spread on an aluminium stub. Each powdered sample was then deposited on the sticky colloidal graphite and excess particles were removed by tapping the stub sideways, followed by blowing with an aerosol duster. Nitrogen adsorption measurements were performed at −196 °C on a Micrometrics ASAP 2020 volumetric adsorption analyzer. Before the analysis, samples were degassed under vacuum at 200 °C for 2 h in the port of the adsorption analyzer.

### 2.4. Electrode Specification

**Figure 1:**
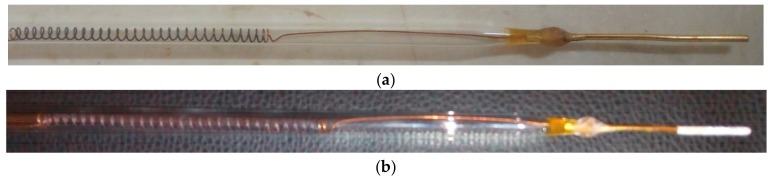
Showing the bare gold electrode (**a**), and after deposition of zeolite X (Na) on the active zone of the surface of the electrode (**b**). The length of the gold is 3 × 40 mm and the active zone is 3 × 5 mm (not drawn to scale).

The electrode was modified to have gold (Au) as the transducer with dimensions 3 mm × 40 mm. The active zone of the gold transducer was 2 mm × 5 mm, to give an active area of 10 mm^2^. Two identical gold electrodes were used to bring the half-cell potential to zero. The surface of the electrode was coated with zeolite X (Na) to enable the adsorption of a biomolecule (bio-receptor) onto its surface and to allow the bio-recognition of the substrate by the zeolite X (Na) modified electrode as shown in Figure 1.

### 2.5. Drop/Dip Coating

To modify the surface of the gold electrode, the following procedure were followed to ensure the deposition of the zeolite on the active zone of the surface of the gold electrode (GE).

0.5 g of zeolite X (Na) was mixed in 0.25 mL of 0.1 mM phosphate buffered saline (PBS) with a pH of 7.4 to form a paste. The active area of the pair of electrodes were dipped in the paste for a maximum of 10 min to enable the adhesion of the paste onto the surface of the electrode under room temperature. The surface modified electrode was placed on a heat block set at 150 °C for 5 min to dehydrate the layer of zeolite on the surface of the gold electrode without changing the orientation of the electrode. This left a thin scale of zeolite uniformly spread over the active zone of the transducer, as shown in Figure 1b.

### 2.6. Immobilization of BSA and Urease 

Zeolite X (Na) modified gold electrodes were dipped into urease and bovine serum albumin (BSA) solutions respectively for optimal absorption. The reference electrode (RE) pre-coated with zeolite X (Na) was dipped into 10% BSA solution. The working electrode (WE), which was also pre-coated with zeolite X (Na) of the same crystallization temperature, was also dipped into 2 mL of 10% urease solution (1 g to 10 g of PBS). The two electrodes were air-dried for 20 min under room temperature in order to maintain the stability of the proteins. The electrodes were then washed off in a working buffer to remove all adsorbed biomolecules from the surface of the electrode. The first layer of zeolite was deposited on the electrodes and later dipped into the urease solution followed by another layer of zeolite dip coating, resulting in a sandwich-like model, after which the electrodes were dipped into the working cell containing 20 mL of 0.1 mM PBS solution and different concentrations of urea. 1 mL of urea solution of 0.1, 0.2, 0.3, 0.4, 0.5, 0.6, 0.7, 0.8, 0.9 and 1.0 M were added to 10 separate 25 mL cells respectively containing 20 mL of 0.1 mM PBS and homogenized with a magnetic stirrer for 3 min. The electrodes were treated similarly for each concentration of urea.

### 2.7. Electrochemical Measurements of the Developed Urea Biosensor

**Figure 2:**
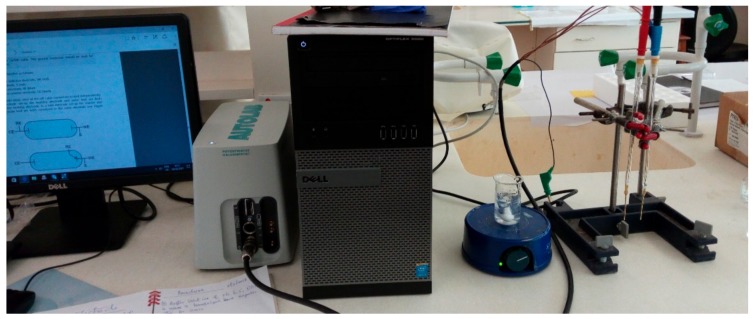
Cyclic voltammetry (CV) measurements were taken using Metrohm AutoLab PGSTAT 204 interfacing a computer with NOVA 2.0 as the control setup and operating software with set parameters.

Cyclic voltammetry (CV) measurements were taken using Metrohm AutoLab PGSTAT 204 interfacing a computer with NOVA 2.0 as the control setup and operating software, with set parameters as displayed in Table 1 and the working setup as displayed in Figure 2. The electrochemical characteristics of the urea biosensor were taken into account as the changes in peak anodic current with reference to the bare electrode, with the anodic current being proportional to the concentration of the analyte. The resulting voltammograms were analysed by the anodic current (oxidation) I_pa_ and the cathodic current (reduction) I_pc_ output. The molar conductance was measured as a function of urea concentration.

## 3. Results and Discussion

**Figure 3:**
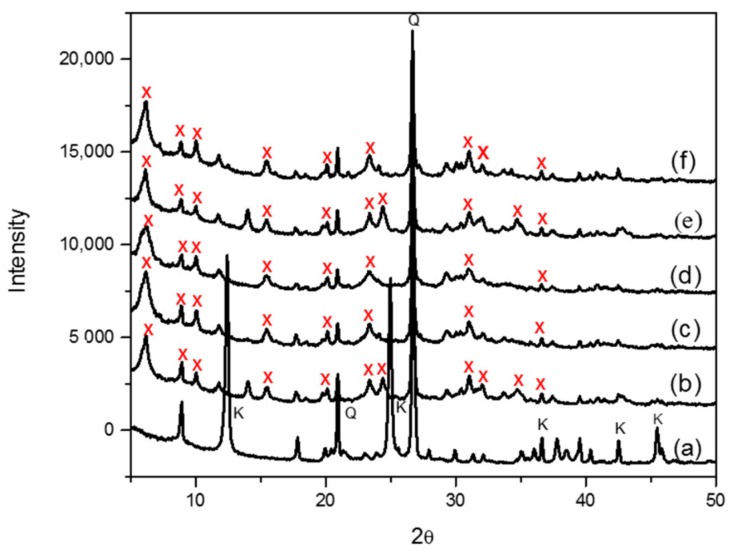
XRD of graphs of zeolite X (Na) synthesized at various temperatures. “K” and “Q” denote kaolinite and β-quartz peaks respectively (**a**). Zeolite X (Na) peaks are indicated in red (**b**–**f**).

**Figure 4:**
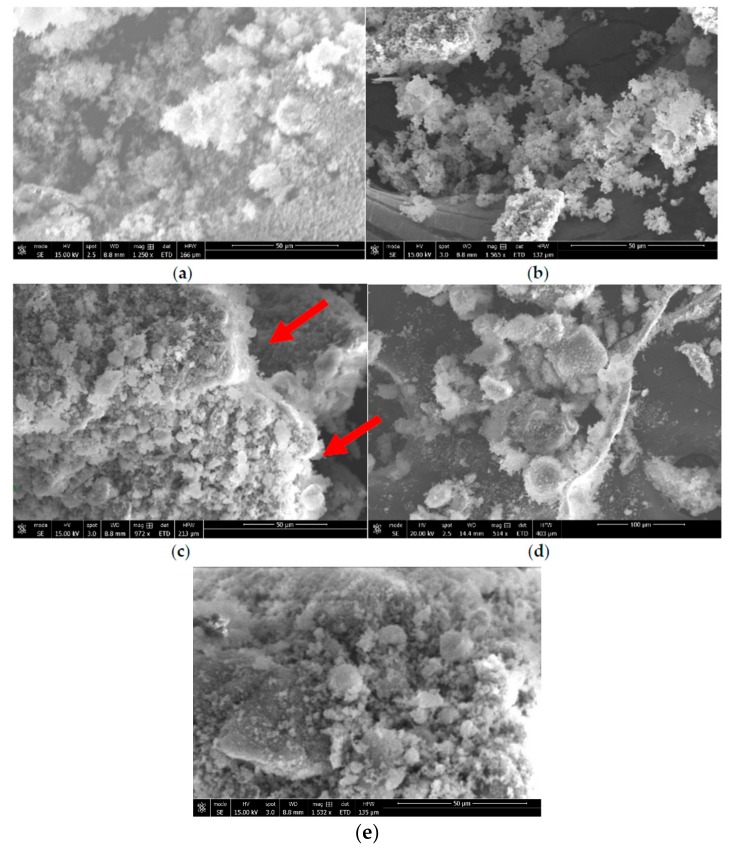
Scanning electron microscopy images of zeolite X (Na) synthesized at (**a**) 60 °C, (**b**) 70 °C, (**c**) 80 °C, (**d**) 90 °C and (**e**) 100 °C at 514–1565× magnification. Scale bar = 50–100 micron.

The synthesis of zeolite X (Na) from kaolin was carried out as a function of the crystallization temperature, based on established methodologies from literature. The crystal phase identification of the individual materials carried out by X-ray diffraction-matching from the X-ray machine and the identified peaks were also validated from the collection of simulated XRD powder patterns for zeolites. As shown in Figure 3, the signature peaks labelled as “K” and “Q” denote kaolinite and β-quartz peaks respectively, typical of kaolin XRD diffraction patterns (Figure 3a). After calcining and undergoing fusion before hydrothermal reaction from 60 °C to 100 °C, three kaolinite peaks at position 9, 12.5 and 25 completely disappeared, with new additional peaks formed as indicated by “X” (Figure 3b–f). All of the peaks labelled “X” are confirmed signature peaks of zeolite X (Na). The peak intensities of zeolite X (Na) were rather low, and this might be due to the formation of a small fraction of a crystalline phase sitting on an amorphous material; nonetheless, all the crystallization temperatures produced zeolite X (Na). First, the SEM results of zeolite X (Na) indicate that there is significant amount of the amorphous phase, which reflects the results obtained from the low intensity XRD spectra. However, with regards to the morphological arrangement of particles, zeolite X (Na) synthesized at 80 and 100 °C revealed well-defined crevices (Figure 4c,e).

**Figure 5:**
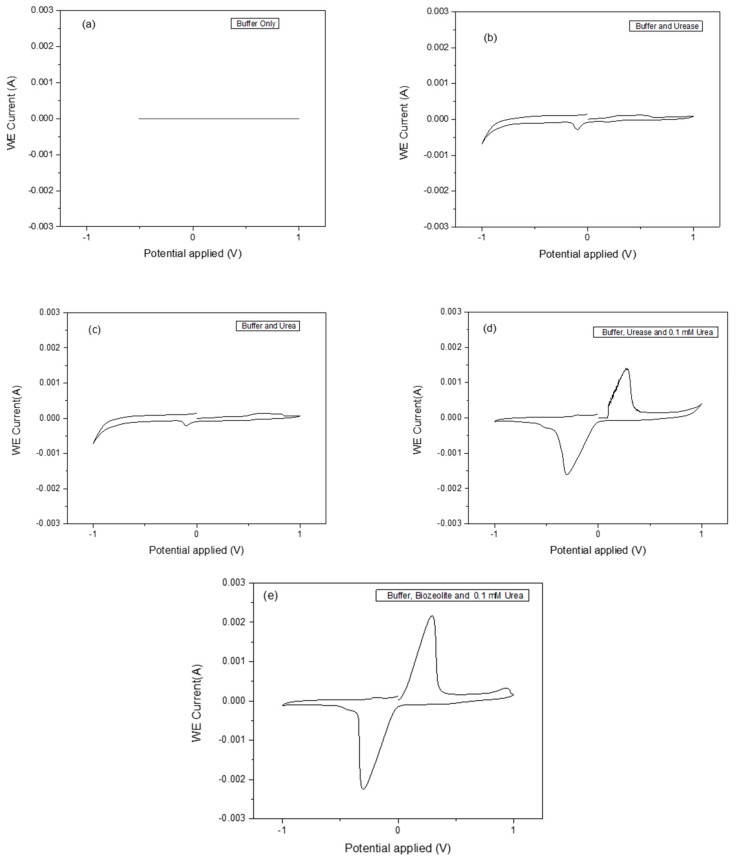
Cyclic voltammogram profiles of the analytical response to (**a**) buffer only, (**b**) buffer and urease, (**c**) buffer and urea, (**d**) buffer, urease and 0.1 mM urea and (**e**) buffer, biozeolite and 0.1 mM urea.

Cyclic voltammetry (CV) is an important and easy-to-use analytical tool to monitor a charge transfer on an electrode surface through the electron transfer process. Urease was chosen due to its stability and has been widely applied in the fabrication of biosensors. The functioning of the urease biosensor is based on the reaction shown in Equation (1), in which the cleavage of urea into ammonium (NH4^+^) ions is as a result of proton consumption results in charge transfer. Different methods for zeolite attachment to the transducer surface were used, and dip coating with heating at 100 °C turned out to be the most suitable method. The parameter-sets in Table 1 were used to measure CV signals as a result of the catalysis of the urea by the immobilized urease enzyme. The analytical responses as measured by the cyclic voltammogram profiles for buffer only, buffer and urease, buffer and urea, buffer, urease and 0.1 mM urea, and buffer, bio-zeolite and 0.1 mM urea are shown in Figure 5a–e. The zeolite X (Na) material synthesized at 80 and 100 °C shows a better response than those synthesized at 60, 70 and 90 °C. As can be seen in Figure 5e, a pair of well-defined, reversible peaks were observed. These peaks can be attributed to the oxidation and reduction of the enzymatically produced NH_4_^+^ ions. It was observed that the reaction rate of the bio-zeolite modified electrode was faster compared to the urease alone using the same amount of urea substrate.

**Figure 6:**
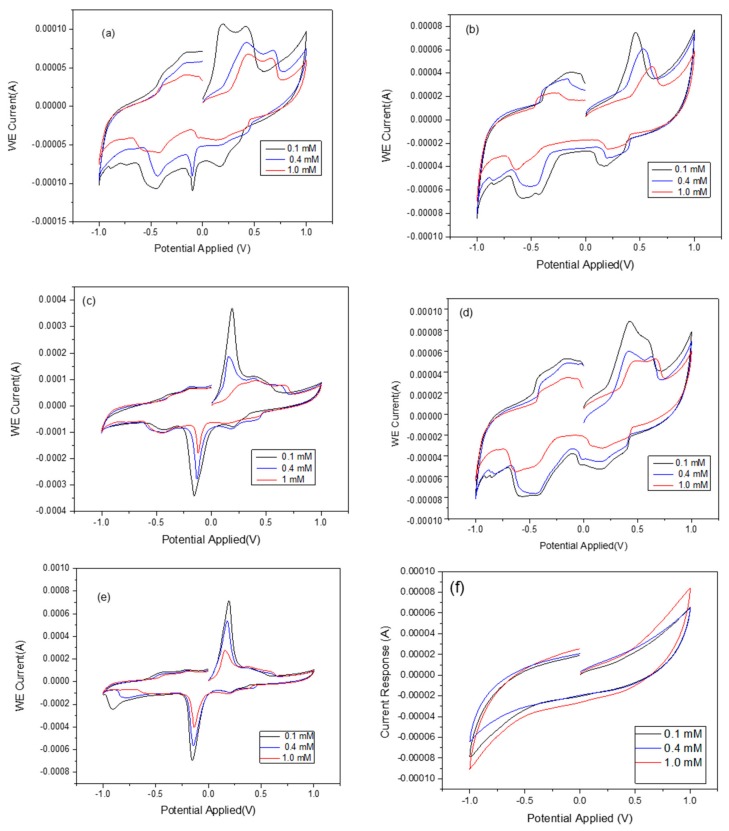
Cyclic voltammograms of the stepwise modification of the GCE electrode with zeolite X (Na) synthesized at (**a**) 60 °C, (**b**) 70 °C, (**c**) 80 °C, (**d**) 90 °C, (**e**) 100 °C and (**f**) Kaolinite only. The colour codes represent different concentrations of urea. The analytical response to different concentration is also shown with different colours.

The effects of zeolite X (Na) based on the crystallization temperature and the analytical response of the biosensor was investigated and the results compared with the raw kaolin. The typical CV responses of zeolite X (Na) 60 °C, zeolite X (Na) 70 °C, zeolite X (Na) 80 °C, zeolite X (Na) 90 °C, zeolite X (Na) 100 °C and pure kaolin as a function of urea concentration are shown in Figure 6. As seen in Figure 6a,b,d, there is a poor response of the biosensor at these crystallization temperatures, with the rate of response being erratic with the zeolite X (Na) synthesized at 60, 70 and 90 °C compared to zeolite X (Na) 80 °C and zeolite X (Na) 100 °C (Figure 6c,e). When compared with the cyclic voltammogram of the pure kaolin, zeolite X (Na) 80 and 100 °C revealed unique analytical responses, indicating that the response is due to the synthesized zeolite rather than the amorphous phase. However, the authors cannot explain why there were no unique cyclic voltammograms at low crystallization temperatures even though the XRD spectra confirmed the formation zeolite X (Na). The cyclic voltammetry reveals oxidation and reduction peaks that indicate the successful immobilization of urease and substrate within the pore network in zeolite X (Na) 80 °C and zeolite X (Na) 100 °C. To confirm this assertion, N_2_ adsorption and desorption measurements were undertaken at 77 K and the results are displayed in Table 2. The zeolite X (Na) 60 °C, zeolite X (Na) 70 °C and zeolite X (Na) 90 °C have comparable surface areas of 64, 68 and 113 m^2^/g respectively, with an average pore size of 2 nm. The materials synthesized at 80 and 100 °C revealed a larger surface area, 295 and 389 m^2^/g, respectively, with an average pore size of 4.5 nm, indicating that zeolite X (Na) 80 °C and zeolite X (Na) 100 °C can accommodate the urease and urea. There is no clear explanation as to why zeolite X (Na) 90 °C did not perform as well as zeolite X (Na) 80 °C and zeolite X (Na) 100 °C. These results are consistent with studies from poly(vinyl alcohol)-modified sol-gel materials, graphene, and gold nanoparticles, as well as zeolites for fabricating a urea biosensor, demonstrated by Doong’s and other groups [29,30,31]. The EDX data in Table 2 revealed that the Si/Al ratio is fairly constant, which is obvious because no additional source of Si and Al are introduced into the material. The estimation of particle size using Shearer’s equation as well as other analytical tools also showed no significant differences in particle size as a function of the crystallization temperature and, therefore, the observed differences in the BET results among zeolite X (Na) are due to the effects of the crystallization temperature and other factors such as pH variations and different ionic concentrations.

**Figure 7:**
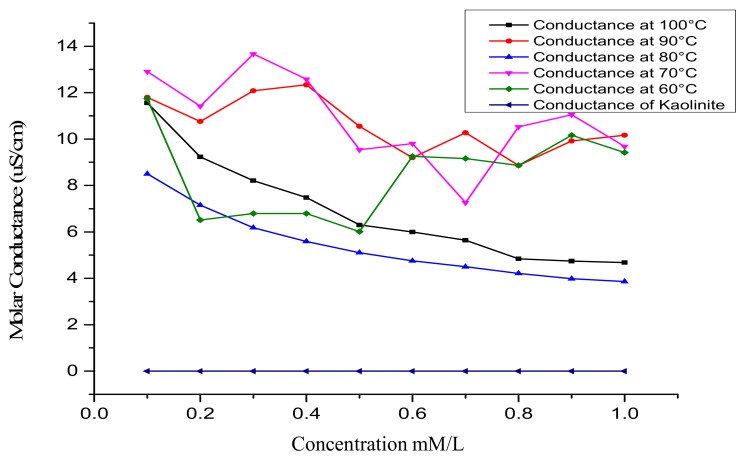
Molar conductance curves of kaolin and zeolite X (Na) modified electrodes and the response of the biosensor as a function of urea concentration.

**Figure 8:**
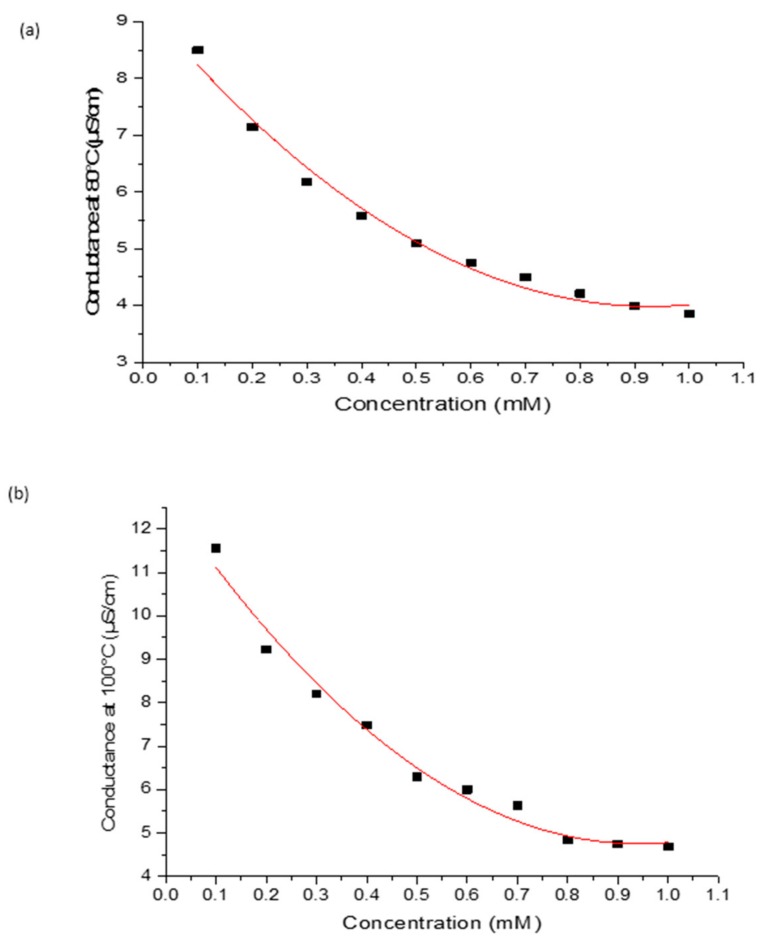
Regression analysis of (**a**) zeolite X (Na) 80 °C and (**b**) zeolite X (Na) 100 °C. Adjusted R-squared were calculated based on seven degrees of freedom.

As shown in Figure 7, the molar conductance of kaolin and the developed bio-zeolites at different crystallization temperatures were measured as a function of urea concentration. First, the kaolin modified electrode showed no response as a function of urea concentration, as indicated by the flat line. The random response of the zeolite X (Na) 60 °C, zeolite X (Na) 70 °C, and zeolite X (Na) 90 °C modified electrode with respect to urea concentration is attributed to the fact that the morphology of the material is not well defined; this results in the inefficient adsorption of the urease on the material. The sensitivity of the urea biosensor at these temperatures is high but inconsequential due to the heterogeneous nature of the pore structures. Regression analysis revealed R^2^ values of 0.193, 0.299, 0.984, 0.350 and 0.980 for zeolite X (Na) 60 °C, zeolite X (Na) 70 °C, zeolite X (Na) 80 °C, zeolite X (Na) 90 °C and zeolite X (Na) 100 °C respectively. In the case of zeolite X (Na) 80 °C and zeolite X (Na) 100 °C, there is a linear correlation due to the creation of a homogenous active surface area as a result of the formation of well-defined surface properties. On the basis of the linear range of 0.1–0.8 unit and 0.1–0.6 mM of the curves at 80 and 100 °C in Figure 8, we speculate that the material properties are influenced by parameters such as the crystallization temperature and pH variations. These might have influenced the conductance of zeolite X (Na) 100 °C modified biosensors compared to zeolite X (Na) 80 °C, but the regression coefficient prefers the former over the later.

In this work, the only parameter that was adjusted was the crystallization temperature; therefore, we preclude that this parameter is a critical determinant of the material properties which could influence enzyme immobilization. Certain temperatures will produce zeolites with good adsorbing characteristics with enhanced analytical responses, such as good detection limits and linearity in response, which are key for developing good biosensors. As a control, we compared the results with the free urease and found that the sensitivity of the immobilized enzyme was higher. It is important to note that the pure kaolin did not exhibit any analytical response. This work therefore seeks to exploit natural materials for the development of biosensors. It is also worth noting that all the experiments were conducted without electrolytic (charged electrolytes) additives; thus, conductive electrolytes such as poly(3,4-ethylene dioxythiophene), PEDOT, NaCl and KCl could be used to modify the zeolites in order to optimize their conductance without the use of electrodes.

## 4. Conclusions

In this study, the influence of temperature on the surface morphology, area and pore size of in-house synthesized zeolites from natural sources, as well as the ability of the zeolites to be employed as carriers for enzyme immobilization, was thoroughly investigated. Pore sizes and pore volumes of zeolite X (Na) 60 °C, zeolite X (Na) 70 °C and zeolite X (Na) 90 °C were comparable and ranged from 1.92 nm to 2.45 nm and 0.012 cm^3^/g–0.061 cm^3^/g, respectively, while that of zeolite X (Na) 80 °C and zeolite X (Na) 100 °C were dramatically increased to 4.21 nm, 0.762 cm^3^/g and 4.92 nm, 0.837 cm^3^/g, respectively. The electroanalytical performance of an immobilized model enzyme—urease—between two mono layers of zeolite X (Na) was also carried out, and the results showed well-defined, reversible redox peaks associated with the enzymatically produced NH_4_^+^ ion, at zeolite X (Na) 80 °C and zeolite X (Na) 100 °C; this indicates that the urease was stably immobilized within the two mono layers of the zeolites. The molar conductance of these zeolites with the immobilized urease was measured as a function of urea concentration and gave an average polynomial regression fit of 0.948. The findings in this study suggest that crystallization temperature influences the morphology of the zeolite, which consequently affects the adsorbed enzyme on the material surface. In future studies, biosensors based on zeolites will be investigated for their stability and selectivity, which is a requirement for biomedical applications.

## Figures and Tables

**Table 1:** The parameter-set used to generate the cyclic voltammetry measurements.

Parameter	Value	Unit
Step Voltage	0.00244	Incremental V
Start Potential	0	V (volts)
Upper Vertex Potential	−1	V_ref_
Low Vertex Potential	1	V_ref_
Scan Rate	100	mV/s
Working electrode (WE) Current	1 mA–100 nA	A

**Table 2:** Characteristics of zeolite synthesized at different crystallization temperatures.

Zeolite	Si:Al Ratio	Pore Diameter (nm)	Specific Surface Area (m^2^/g)	Pore Volume (cm^3^/g)	Average Particle Size (nm)
Zeolite X (Na) 60 °C	1.3	2.23	64.32	0.012	44.32
Zeolite X (Na) 70 °C	1.0	2.45	67.77	0.017	39.77
Zeolite X (Na) 80 °C	1.2	4.21	295.25	0.762	35.30
Zeolite X (Na) 90 °C	1.3	1.92	113.14	0.061	36.10
Zeolite X (Na) 100 °C	1.2	4.92	389.43	0.837	44.30
